# Risk of early death after acute leukemia diagnosis among adolescents and young adults

**DOI:** 10.1093/jncics/pkaf065

**Published:** 2025-06-20

**Authors:** Amy M Berkman, Clark R Andersen, Vidya Puthenpura, Nicholas J Short, Kelly Merriman, Mahesh Swaminathan, Branko Cuglievan, David McCall, Courtney DiNardo, Cesar Nunez, Nitin Jain, Tapan Kadia, Ghayas Issa, Amber Gibson, Miriam B Garcia, J Andrew Livingston, Susan Parsons, Michelle A T Hildebrandt, Michael E Roth

**Affiliations:** Department of Oncology, St. Jude Children’s Research Hospital, Memphis, TN 38105, United States; Department of Biostatistics, The University of Texas MD Anderson Cancer Center, Houston, TX 77030, United States; Section of Pediatric Hematology and Oncology, Department of Pediatrics, Yale School of Medicine, New Haven, CT 06510, United States; Department of Leukemia, The University of Texas MD Anderson Cancer Center, Houston, TX 77030, United States; Department of Cancer Registry, The University of Texas MD Anderson Cancer Center, Houston, TX 77030, United States; Department of Leukemia, The University of Texas MD Anderson Cancer Center, Houston, TX 77030, United States; Department of Pediatrics, The University of Texas MD Anderson Cancer Center, Houston, TX 77030, United States; Department of Pediatrics, The University of Texas MD Anderson Cancer Center, Houston, TX 77030, United States; Department of Leukemia, The University of Texas MD Anderson Cancer Center, Houston, TX 77030, United States; Department of Pediatrics, The University of Texas MD Anderson Cancer Center, Houston, TX 77030, United States; Department of Leukemia, The University of Texas MD Anderson Cancer Center, Houston, TX 77030, United States; Department of Leukemia, The University of Texas MD Anderson Cancer Center, Houston, TX 77030, United States; Department of Leukemia, The University of Texas MD Anderson Cancer Center, Houston, TX 77030, United States; Department of Pediatrics, The University of Texas MD Anderson Cancer Center, Houston, TX 77030, United States; Department of Pediatrics, The University of Texas MD Anderson Cancer Center, Houston, TX 77030, United States; Department of Sarcoma Medical Oncology, The University of Texas MD Anderson Cancer Center, Houston, TX 77030, United States; Division of Hematology/Oncology and Institute for Clinical Research and Health Policy Studies, Tufts Medical Center Cancer Center, Boston, MA 02111, United States; Department of Lymphoma-Myeloma, The University of Texas MD Anderson Cancer Center, Houston, TX 77030, United States; Department of Pediatrics, The University of Texas MD Anderson Cancer Center, Houston, TX 77030, United States

## Abstract

**Background:**

Advances in care have led to improvements in survival for adolescents and young adults (AYAs) diagnosed with cancer; however, the risk of early death remains high for certain cancers, particularly acute leukemias. Risk factors for early death in AYAs diagnosed with acute leukemia have not been well studied.

**Methods:**

The Surveillance, Epidemiology, and End Results registry was used to assess risk of early death (within 2 months of diagnosis) in AYAs diagnosed with acute leukemia (*n* = 16 153). Early death proportion, by year, for AYAs diagnosed between 2006 and 2020 was described. Associations between incidence of early death and age at diagnosis, sex, race and ethnicity, socioeconomic status, rurality, acute leukemia type, and year of diagnosis were evaluated with logistic regression.

**Results:**

Overall, 6.0% of AYAs experienced early death and there was a significant annual decrease in the odds of early death (odds ratio [OR] = 0.96, 95% confidence interval [CI] = 0.95 to 0.98, *P* < .0001) across the study period. Over the entire study period, AYAs diagnosed with acute promyelocytic leukemia (9.6%, 95% CI = 8.4 to 11.1) or other acute leukemias (13.3%, 95% CI = 10.5 to 16.7) had the highest proportion of early death and AYAs diagnosed with T lymphoblastic leukemia/lymphoma had the lowest (2.6%, 95% CI = 1.9 to 3.7). Older age at diagnosis, male sex, and Hispanic ethnicity were all associated with increased risk of early death.

**Conclusions:**

A high proportion of AYAs with acute leukemia experience early death and risk varies by leukemia type and sociodemographic factors. A better understanding of the complex interplay between disease biology and sociodemographic factors is needed to guide risk prediction and prevention.

## Introduction

Although overall survival outcomes for adolescents and young adults (AYAs; aged 15-39 years) diagnosed with cancer have improved over recent decades,^1^ there remains a subset at risk for death soon after diagnosis. Sociodemographic factors including age at diagnosis, race and ethnicity, and neighborhood-level socioeconomic status (SES) have been identified as associated with risk of early death (death within 2 months of diagnosis) among AYAs.^2^ Cancer type is also an important risk factor for early death with AYAs diagnosed with hematologic malignancies at higher risk than those diagnosed with central nervous system or solid tumors. AYAs with acute leukemias are at a particularly high risk for early death. A prior analysis found that those with acute myeloid leukemia (AML) and acute lymphoid leukemia had a 9.4% and 4.3% prevalence of early death during the years 2000-2016, compared with a <1% prevalence of early death for those diagnosed with other common AYA malignancies including thyroid cancer, melanoma, breast cancer, or Hodgkin lymphoma.^2^ As acute leukemias are among the most common cancer types diagnosed in AYAs,^3^ a better understanding of the epidemiology of early death in this population is needed to guide risk prediction and identify opportunities for early intervention to improve survival.

In the current study, we used data from the Surveillance, Epidemiology, and End Results (SEER)-17 database, a population-based registry, to characterize early death in AYAs diagnosed with acute leukemia from 2006 to 2020. We evaluated trends in early death by year as well as assessed the associations between age, sex, race and origin, rurality, neighborhood-level SES, and acute leukemia type with early death, defined as death within 2 months of diagnosis.

## Methods

### Study population

Individuals diagnosed with acute leukemia between the ages of 15 and 39.99 years and between diagnosis years of January 2006 and December 2020 and whose data were available in SEER-17 were included in this retrospective cohort study which was approved by the MD Anderson institutional review board. SEER was started in 1973 and collects demographic, clinical, and outcomes data on all incident cancers within its specified geographic areas.^4^ The population covered has incrementally expanded and SEER-17 contains data from the National Cancer Institute from 17 population-based regional cancer registries across the United States that cover about 28% of the population.^5^ SEER data also include national mortality data from the National Vital Statistics System. SEER ICD-O-3 acute leukemia codes included in the study are shown in Table S1. Acute leukemia diagnoses were categorized by the AYA site recode World Health Organization categorization into AML, B lymphoblastic leukemia/lymphoma, T lymphoblastic leukemia/lymphoma, acute promyelocytic leukemia (APL), Burkitt leukemia, and other acute leukemia. In SEER, B lymphoblastic leukemia and lymphoma are grouped into one category as are T lymphoblastic leukemia and lymphoma. Only primary cancer diagnoses were included.

### Study variables

Early death was defined as death within 2 months of acute leukemia diagnosis date,^2^ reported as 0- or 1-month “survival months” in SEER. Race and origin were defined by the SEER “race and origin” variable as Hispanic (all races), non-Hispanic Asian or Pacific Islander (hereafter, Asian or Pacific Islander), non-Hispanic Black (hereafter, Black), non-Hispanic White (hereafter, White), and other. Rurality was categorized as rural and urban per the variable “rural-urban commuting area-based categorization C (2 categories).” Neighborhood-level SES was categorized into Yost quintiles, a census-tract level index that takes into account median rent, median household income, percentage less than 150% of the poverty line, median house value, percentage unemployed, percentage working class, and education index.^6^ Quintile 1 represents the lowest SES and quintile 5 represents the highest SES.

### Statistical analysis

In cases where there was more than 1 record per patient, analyses used the first record for each patient. Demographics and clinical characteristics were summarized by early death status using mean, standard deviation, median, quartiles, and range, or frequency with percentage. Differences were assessed by 2-sample 2-sided *t*-test and Mann-Whitney test or χ^2^ test. Unadjusted summaries of percentage of patients with early death were summarized by year of diagnosis and by cancer diagnosis with 95% Agresti-Coull confidence intervals.

Variable selection of covariates for the logistic regression model of incidence of early death was based upon the subset selection algorithm,^7^ using the rFSA package, with the optimal model subsequently enhanced to accommodate potential penalized splines over year of diagnosis, with final selection based upon the model with lowest Akaike Information Criterion.^8^ The resulting optimal logistic model was a generalized additive model with logit link relating incidence of early death to age, race and origin, sex, rurality status, Yost quintile, cancer type, and a penalized spline over year of diagnosis.^9^ Addition of interactions between cancer type with age, race and origin, sex, rurality, Yost quintile, and year of diagnosis were explored but did not improve the model over the model without interactions. Differences among the categories of discrete variables were assessed by contrasts with relation to the reference category, with Dunnett-adjusted *P*-values for variables with more than 2 levels. A corresponding generalized linear model without the penalized spline over year of diagnosis was utilized to assess the covariate-adjusted rate of change by year of diagnosis prior to 2020, excluding 2020 data.

Statistical analyses used a 95% level of statistical confidence (per α = 0.05). All statistical analyses were performed using R statistical software version 4.4.1. The “catseyes” package (Andersen 2020) was used to produce Catseye plots.^10^^,^^11^

## Results

### Participant characteristics

The study included 16 153 AYAs diagnosed with cancer between the years 2006 and 2020. Overall, the mean age of participants was 26.6 years (SD = 7.6); the mean age of those without early death was 26.5 years (SD = 7.6) and the mean age of those with early death was 28.4 years (SD = 7.3), respectively (Table 1). The majority of participants in all groups were male and lived in urban areas. White individuals made up the highest proportion of race and origin groups in the overall (41.4%) and no early death groups (41.7%) and Hispanic individuals made up the largest proportion of the early death group (41.6%). In all groups, the most common Yost quintile was 1 (lowest SES) and the most common acute leukemia diagnosis was AML. The mean number of months from diagnosis date to treatment initiation was 0.3 (SD = 1.0) in both the population overall and among AYAs without early death and was 0.1 (SD = 0.3) among AYAs with early death.

**Table 1.** Characteristics of 16 153 AYAs diagnosed with acute leukemia between the years 2006 and 2020 identified in the SEER database overall and by early death status.

Characteristic	Overall (*n* = 16 153) No. (%)	No early death (*n* = 15 178) No. (%)	Early death (*n* = 975) No. (%)	*P* ^b^
**Age at diagnosis, y**				**<.0001**
Mean (SD)	26.6 (7.6)	26.5 (7.6)	28.4 (7.3)	
Median (interquartile range)	26 (20-33)	26 (20-33)	30 (22-35)	
**Sex**				**.001**
Female	7016 (43.4)	6641 (43.8)	375 (38.5)	
Male	9137 (56.6)	8537 (56.2)	600 (61.5)	
**Race and origin**				**.03**
Asian or Pacific Islander	1372 (8.5)	1282 (8.4)	90 (9.2)	
Black	1614 (10.0)	1503 (9.9)	111 (11.4)	
Hispanic (all races)	6284 (38.9)	5878 (38.7)	406 (41.6)	
White	6683 (41.4)	6327 (41.7)	356 (36.5)	
Other	200 (1.2)	188 (1.2)	12 (1.2)	
**Rurality**				.87
Rural	1568 (9.7)	1472 (9.7)	96 (9.8)	
Urban	14 585 (90.3)	13 706 (90.3)	879 (90.2)	
**Yost index quintile^a^**				**.04**
1 (Lowest SES)	3642 (22.6)	3389 (22.3)	254 (26.1)	
2	3199 (19.8)	2998 (19.8)	201 (20.6)	
3	2979 (18.4)	2803 (18.5)	176 (18.1)	
4	3087 (19.1)	2920 (19.2)	167 (17.1)	
5 (Highest SES)	3245 (20.1)	3068 (20.2)	177 (18.2)	
**Acute leukemia type**				**<.001**
B lymphoblastic leukemia/lymphoma	5905 (36.6)	5696 (37.5)	209 (21.4)	
AML	6409 (39.7)	5928 (39.1)	481 (49.3)	
APL	1888 (11.7)	1706 (11.2)	182 (18.7)	
Burkitt leukemia	268 (1.7)	259 (1.7)	9 (0.9)	
T lymphoblastic leukemia/lymphoma	1218 (7.5)	1186 (7.8)	32 (3.3)	
Other acute leukemia	465 (2.9)	403 (2.7)	62 (6.4)	
**Months from diagnosis to treatment**				
Mean (SD)	0.3 (1.0)	0.3 (1.0)	0.1 (0.3)	**<.0001**

Abbreviations: AML = acute myeloid leukemia; APL = acute promyelocytic leukemia; AYA = adolescents and young adults; SEER = Surveillance, Epidemiology, and End Results; SES = socioeconomic status.

a The Yost index is a census-tract level index that takes into account median rent, median household income, percentage less than 150% of the poverty line, median house value, percentage unemployed, percentage working class, and education index.

b Bolded values indicate *P*-values reaching statistical significance.

### Percentages of early death

**Figure 1.**
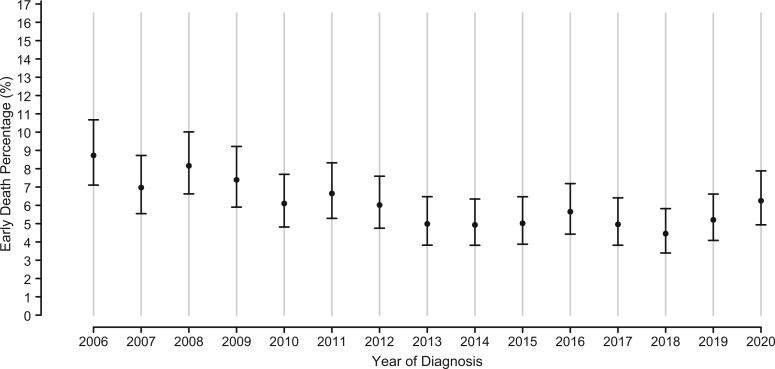
Early death percentage in adolescents and young adults with acute leukemia by year of diagnosis. Bars represent 95% confidence intervals.

**Figure 2.**
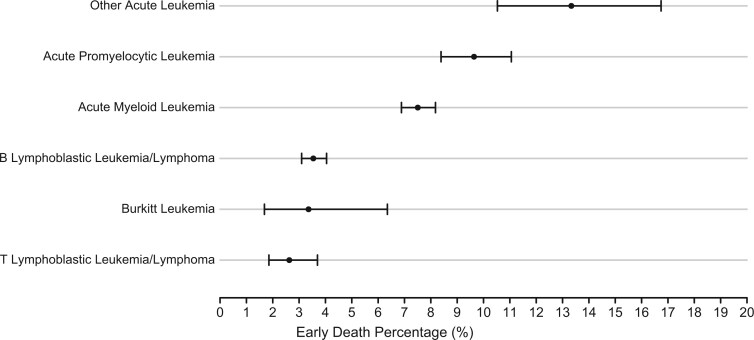
Early death percentage in adolescents and young adults with acute leukemia by leukemia type. Bars represent 95% CIs.

Overall, 6.0% of the AYAs diagnosed with acute leukemia from 2006 to 2020 experienced early death. In 2006, the proportion of AYAs diagnosed with acute leukemia who had early death was 8.7% (95% confidence interval [CI] = 7.1 to 10.7) and by 2018 the proportion had declined to 4.5% (95% CI = 3.7 to 6.2) before rising to 5.2% (95% CI = 4.1 to 6.6) in 2019 and 6.3% (95% CI = 4.9 to 7.9) in 2020 (Figure 1, Table S2). A covariate-adjusted logistic regression model without penalized spline shows a significant annual decrease in the odds of early death (odds ratio [OR] = 0.96, 95% CI = 0.95 to 0.98, *P* < .0001) across the study period. Early death varied by acute leukemia diagnosis. AYAs diagnosed with APL (9.6%, 95% CI = 8.4 to 11.1) and other acute leukemias (13.3%, 95% CI = 10.5 to 16.7) had the highest proportion of early death over the study period and AYAs diagnosed with T lymphoblastic leukemia/lymphoma had the lowest proportion of early death (2.6%, 95% CI = 1.9 to 3.7) (Figure 2).

### Predictors of early death

**Figure 3.**
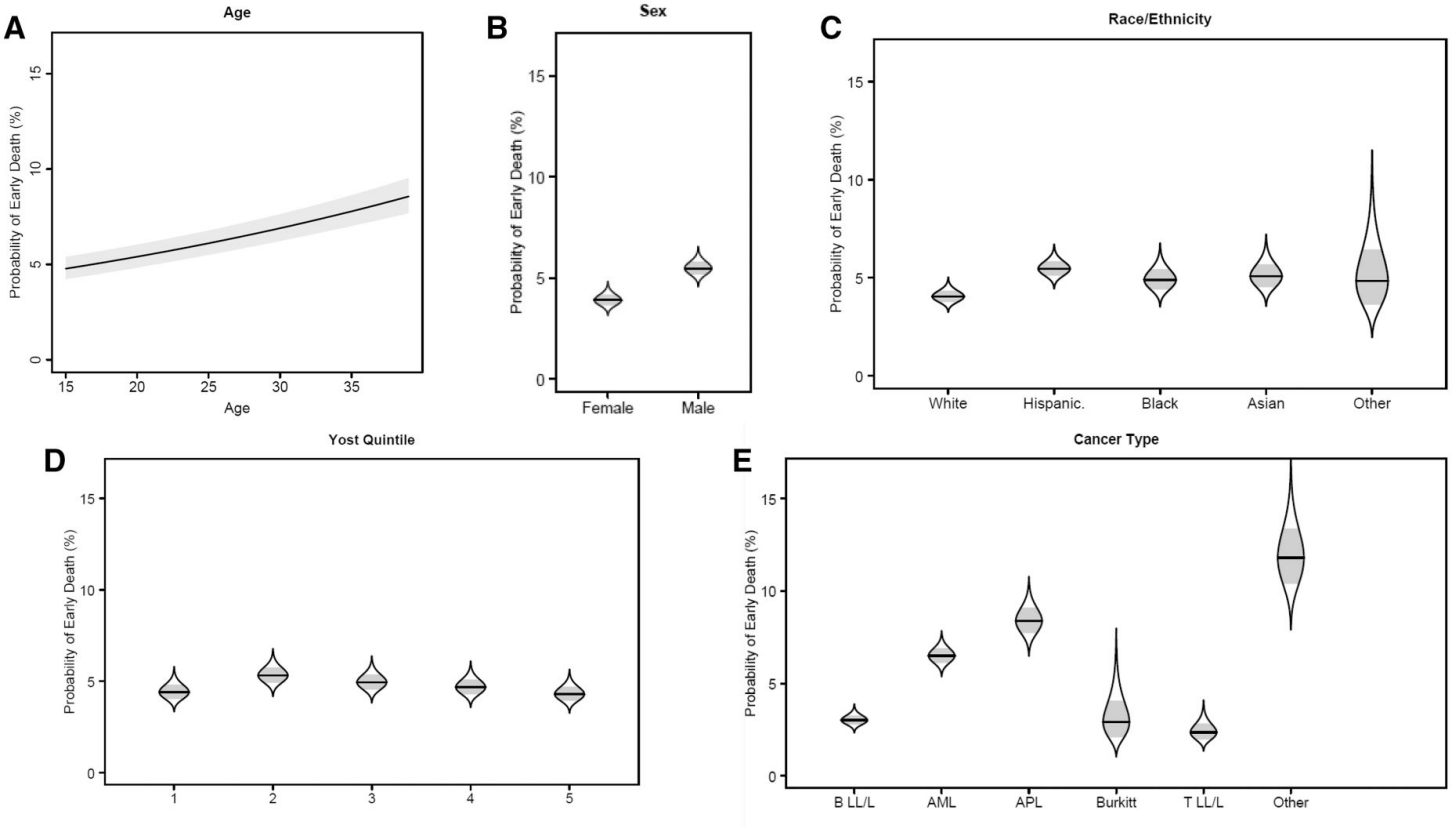
Model-adjusted odds of early death in adolescents and young adults with acute leukemia by (A) age, (B) sex, (C) race and ethnicity, (D) SES (measured by Yost Index quintile), and (E) leukemia type. (A) shows the model-adjusted probability of early death by age at diagnosis with shaded standard error intervals. (B-E) show catseye plots representing the normal distributions of the model-adjusted means with shaded standard error intervals.

Older age at diagnosis (as a continuous variable) was associated with higher odds of early death (Odds Ratio [OR]:1.03, 95% CI: 1.02-1.04 for each unit increase) among AYAs diagnosed with acute leukemia (Table 2, Figure 3A). Males had 43% higher odds of early death than females (OR: 1.43, 95% CI: 1.25-1.64, Figure 3B). Hispanic AYAs had higher odds of early death compared to White AYAs (OR: 1.37, 95% CI: 1.17-1.61, Figure 3C), while there were no significant differences between White and Black, Asian or Pacific Islander AYAs, or AYAs of other races/ethnicities. While not reaching statistical significance, AYAs with the lowest neighborhood level SES had higher odds of early death than those with the highest neighborhood level SES (OR: 1.22, 95% CI: 0.98-1.52, Figure 3D). Finally, compared to AYAs with B lymphoblastic leukemia/lymphoma, those with AML (OR: 2.24, 95% CI: 1.88-2.66), APL (OR: 2.94, 95% CI: 2.38-3.64), and acute leukemias classified as “other” (OR: 4.30, 95% CI: 3.17-5.83) had higher odds of early death (Figure 3E).

**Table 2.** Model-adjusted odds ratios of early death among 16 153 AYAs diagnosed with acute leukemia between 2006 and 2020 identified in the SEER database.

Characteristic	Odds ratio (95% CI)	*P* ^b^
**Age at diagnosis**		
Unit change	1.03 (1.02 to 1.04)	**<.0001**
**Sex**		
Female	1.0	
Male	1.43 (1.25 to 1.64)	**<.0001**
**Race and origin**		
White	1.0	
Asian and Pacific Islander	1.27 (1.00 to 1.62)	.17
Black	1.22 (0.97 to 1.54)	.27
Hispanic	1.37 (1.17 to 1.61)	**<.001**
Other	1.21 (0.66 to 2.20)	.89
**Rurality**		
Urban	1.0	
Rural	1.01 (0.80 to 127)	.96
**Yost index quintile** ^a^		
1	1.22 (0.98 to 1.52)	.23
2	1.13 (0.91 to 1.41)	.63
3	1.07 (0.85 to 1.33)	.91
4	0.98 (0.78 to 1.22)	.99
5	1.0	
**Acute leukemia type**		
B lymphoblastic leukemia/lymphoma	1.0	
AML	2.24 (1.88 to 2.66)	**<.0001**
APL	2.94 (2.38 to 3.64)	**<.0001**
Burkitt leukemia	0.97 (0.49 to 1.91)	1.0
T lymphoblastic leukemia/lymphoma	0.78 (0.53 to 1.14)	.55
Other acute leukemia	4.30 (3.17 to 5.83)	**<.0001**

Abbreviations: AML = acute myeloid leukemia; APL = acute promyelocytic leukemia; AYA = adolescents and young adults; CI = confidence interval; SEER = Surveillance, Epidemiology, and End Results.

a The Yost index is a census-tract level index that takes into account median rent, median household income, percentage less than 150% of the poverty line, median house value, percentage unemployed, percentage working class, and education index.

b Bolded values indicate *P*-values reaching statistical significance.

## Discussion

The incidence of cancer in AYAs is increasing, and, although 5-year survival rates have improved overall, certain groups of AYAs, including those diagnosed with acute leukemia, remain at risk for early death within the first 2 months of diagnosis.^2^^,^^3^ We found that, among AYAs diagnosed with acute leukemia, the percentage of early death was higher among those diagnosed with APL, AML, and other acute leukemias and lower among those diagnosed with B lymphoblastic leukemia/lymphoma, T lymphoblastic leukemia/lymphoma, and Burkitt leukemia. Additionally, AYAs diagnosed at older ages, males, and AYAs with Hispanic ethnicity were at increased risk of early death compared with AYAs diagnosed at younger ages, females, and White AYAs, respectively. Importantly, we found that the percentage of early deaths per year was decreasing over the study period.

We found that AYAs diagnosed with AML, APL, and other acute leukemias had the highest prevalences of early death over the study period at 7.5%, 9.6%, and 13.3%, respectively. Treatment-related toxicities, particularly infectious complications due to treatment-induced neutropenia and immune dysfunction, remain a common cause of death during induction therapy for acute leukemias in general.^12-15^ Prior studies have found that bleeding, possibly due to hyperleukocytosis and sludging and/or direct damage to the endothelium secondary to the migration of leukemic cells, is a common cause of early death in patients with AML.^16^ Hemorrhagic complications due to coagulopathies such as disseminated intravascular coagulation are also a common cause of early death in patients diagnosed with APL.^17^ Differentiation syndrome, a systemic inflammatory response to treatment with all-trans retinoic acid and arsenic trioxide leading to end organ damage including hypotension, interstitial pulmonary infiltrates, and acute renal failure is another common cause of early death in patients with APL.^18^ Acute leukemias classified as “other” are rare and may include poorly differentiated leukemias of ambiguous lineage.^19^^,^^20^ These are often difficult to diagnose and highly aggressive,^21^ which may also contribute to the high prevalence of early death. It is also possible a portion of AYAs with early death died prior to definitive leukemia diagnosis and thus were classified as having “other” leukemia.

Sociodemographic factors were also associated with risk of early death among AYAs diagnosed with acute leukemia. For each year of increasing age at diagnosis, AYAs had a 3% higher risk of early death. This is consistent with studies among both childhood and older adults diagnosed with acute leukemia^15^^,^^22^ but has not been well studied in AYAs. Older AYAs may be more susceptible to treatment-related toxicity,^23^ potentially contributing to an increased risk of early death. Consistent with early death data including all AYA cancer diagnoses and overall survival data in AYAs with acute leukemia,^2^^,^^24^^,^^25^ male AYAs with acute leukemia were at higher risk for early death than females. Research investigating sex-associated differences in genetic markers of acute leukemias is emerging as potentially contributing to treatment response,^26^ and these genetic differences could also play a role in susceptibility to severe treatment complications. Social factors related to sex, such as sex-associated differences in treatment adherence, could not be explored in SEER-17.

AYAs with Hispanic ethnicity were at higher risk of early death than White AYAs, whereas there were no differences in risk of early death between Black, Asian and Pacific Islander, or AYAs with other race or ethnicity and White AYAs. Hispanic individuals are more likely than White individuals with acute leukemia have high-risk cytogenetics,^27^^,^^28^ although how this imparts increased risk of early death has not been studied. Clinical factors may also play a role in disparities by ethnicity. For example, Hispanic individuals with APL are more likely to have high risk disease, higher white blood cell count, and coagulopathies at diagnosis compared with non-Hispanic individuals with APL and have a higher burden of toxicities during induction therapy.^29^ Additionally, there are racial and ethnic disparities in early diagnosis and clinical trial enrollment among individuals with acute leukemia, and these likely contribute to risk of early death.^30^^,^^31^ Although we did not see differences in early death in Black compared with White AYAs with acute leukemia, prior studies have found worse outcomes, including higher risk of early death in Black AYAs with AML, specifically.^25^^,^^32^ Finally, although not reaching statistical significance, AYAs living in areas with the lowest SES measures had a higher risk of early death than AYAs living in areas with the highest SES measures. Lower SES is associated with higher stage of disease at diagnosis among AYAs with cancer and lower rates of treatment initiation in adult cancer patients,^33^^,^^34^ and these factors may also contribute to an increased risk of early death.

Importantly, although there was variation in early death by year of diagnosis, we found an overall trend of decreasing early death over the study period. The routine use of antimicrobial prophylaxis during induction therapy has likely resulted in lowering infection-related early death in AYAs with acute leukemia.^35-37^ There have not yet been similar widespread interventions that have successfully targeted other causes of early death such as hemorrhage or differentiation syndrome, though early work has shown promise. Specifically, a recent trial that, upon suspected APL presentation at a community hospital, connected community physicians with local APL experts for work-up, management, and treatment recommendations during the first 2 weeks after presentation resulted in a significant decrease in early deaths.^38^ Recent work has also aimed to risk stratify and standardize the management of the coagulopathy associated with APL and its treatment; however, determining optimal preventative and therapeutic strategies remains a challenge.^39^

There are limitations that should be considered when interpreting the results of this study. Although SEER is considered an authoritative source of population-level cancer data in the US, certain variables of potential interest are unavailable within the dataset. These include detailed information on therapeutic exposures, clinical factors at presentation, and initial diagnosis and treatment setting (ie, academic medical center vs community hospital) thereby limiting our ability to determine how these may have been impacted risk of early death. Detailed clinical and biological data are also unavailable, highlighting the need for future studies using clinical trial or institutional datasets to evaluate the association of these factors with early death in AYAs with acute leukemia. Similarly, exact cause of death is unknown. SEER reports diagnosis date to the nearest year and survival data in integer months, thus it is possible that some AYAs with acute leukemia died on the day of diagnosis and these cases would have been considered in our study as early death events even though they may represent different clinical populations than those who died within the first 2 months of diagnosis. Additionally, it is known that insurance status is associated with survival outcomes among AYAs with cancer, including early death.^2^ However, SEER stopped reporting their insurance variable with the 2019 data submission;^40^^,^^41^ therefore, we were unable to evaluate the association of insurance status with risk of early death among AYAs diagnosed with acute leukemia.

In conclusion, we demonstrated a high risk of early death in AYAs diagnosed with acute leukemia, particularly among those with APL, AML, and other acute leukemias. Sociodemographic factors including older age, male sex, and Hispanic ethnicity were associated with higher risk of early death. A better understanding of the complex interplay between disease biology and social drivers of health is needed to guide early death risk prediction and prevention. Finally, we found an overall trend of decreasing early death over the study period. This is a promising finding that likely reflects improvements in supportive care for AYAs with acute leukemia; however, our data demonstrate that there is still work to be done in the optimization and standardization of early supportive care, particularly for those at risk for hemorrhagic complications.

## Supplementary Material

pkaf065_Supplementary_Data

## Data Availability

Data used for this study were obtained from the publicly available Surveillance, Epidemiology, and End Results Program database (https://seer.cancer.gov/). Access to SEER is granted through a data request and upon agreement to SEER data agreements and limitations.
