# Supplementary material for: Risk of early death after acute leukemia diagnosis among adolescents and young adults
Source: JNCI Cancer Spectr. 2025 Jun 20;9(4):pkaf065. doi: 10.1093/jncics/pkaf065 (PMC12231595; doi:10.1093/jncics/pkaf065)
Supplement: pkaf065_Supplementary_Data [file pkaf065_supplementary_data.docx]

**Supplementary Materials**

**Supplementary Tables**

**Table S1**. Acute Leukemia Diagnoses from Surveillance, Epidemiology, and End Results ICD-O-3 Codes

| **ICD-O-3 Code** | **Name** | **ICD-O-3 Recode** |
| --- | --- | --- |
| 9801/3 | Acute undifferentiated leukemia | Other Acute Leukemia |
| 9805/3 | Acute biphenotypic leukemia | Other Acute Leukemia |
| 9806/3 | Mixed pheno acute leuk w/t(9;22)(q34;q11.2);BCR-ABL1 | Other Acute Leukemia |
| 9807/3 | Mixed phenotype acute leukemia with t(v;11q23); MLL rearranged | Other Acute Leukemia |
| 9808/3 | Mixed phenotype acute leukemia, B/myeloid, NOS | Other Acute Leukemia |
| 9809/3 | Mixed-phenotype acute leukemia, T/myeloid, NOS | Other Acute Leukemia |
| 9811/3 | B-lymphoblastic leukemia/lymphoma, NOS | B Lymphoblastic Leukemia/Lymphoma |
| 9812/3 | B lymphobl leuk/lymph w/t(9;22)(q34;q11.2); BCR-ABL1 | B Lymphoblastic Leukemia/Lymphoma |
| 9813/3 | B lymphobl leuk/lymph w/t(v;11q23); MLL rearranged | B Lymphoblastic Leukemia/Lymphoma |
| 9814/3 | B lymphobl leuk/lymph w/t(12;21)(p13;q22);TEL-AML1 | B Lymphoblastic Leukemia/Lymphoma |
| 9815/3 | B lymphoblastic leuk/lymph w/hyperdiploidy | B Lymphoblastic Leukemia/Lymphoma |
| 9816/3 | B lymphobl leuk/lymph w/hypodiploidy (hypodip ALL) | B Lymphoblastic Leukemia/Lymphoma |
| 9817/3 | B lymphobl leuk/lymph w/t(5;14)(q31;q32); IL3-IGH | B Lymphoblastic Leukemia/Lymphoma |
| 9818/3 | B lymphobl leuk/lymph w/t(1;19)(q23;p13.3); E2A PBX1 | B Lymphoblastic Leukemia/Lymphoma |
| 9826/3 | Burkitt cell leukemia | Burkitt Leukemia |
| 9827/3 | Adult T-cell leukemia/lymphoma | T Lymphoblastic Leukemia/Lymphoma |
| 9831/3 | T-cell large granular lymphocytic leukemia | T Lymphoblastic Leukemia/Lymphoma |
| 9833/3 | B-cell prolymphocytic leukemia | B Lymphoblastic Leukemia/Lymphoma |
| 9834/3 | T-cell prolymphocytic leukemia | T Lymphoblastic Leukemia/Lymphoma |
| 9835/3 | Precursor cell lymphoblastic leukemia, NOS | B Lymphoblastic Leukemia/Lymphoma |
| 9836/3 | Precursor B-cell lymphoblastic leukemia | B Lymphoblastic Leukemia/Lymphoma |
| 9837/3 | T lymphoblastic leukemia/lymphoma | T Lymphoblastic Leukemia/Lymphoma |
| 9840/3 | Acute erythroid leukemia | Acute Myeloid Leukemia |
| 9860/3 | Myeloid leukemia, NOS | Acute Myeloid Leukemia |
| 9861/3 | Acute myeloid leukemia, NOS | Acute Myeloid Leukemia |
| 9865/3 | Acute myeloid leukemia with t(6;9)(p23;q34);DEK-NUP214 | Acute Myeloid Leukemia |
| 9866/3 | Acute promyelocytic leukemia (AML with t(15;17)(q22;q12)) PML/RARA | Acute Promyelocytic Leukemia |
| 9867/3 | Acute myelomonocytic leukemia | Acute Myeloid Leukemia |
| 9869/3 | Acute myeloid leuk. inv(3)(q21;q26.2) or t(3;3)(q21;q26.2); RPN1-EVI1 | Acute Myeloid Leukemia |
| 9870/3 | Acute basophilic leukemia | Acute Myeloid Leukemia |
| 9871/2 | AML with inv(16)(p13.1q22) or t(16;16)(p13.1;q22), CBFB-MYH11 | Acute Myeloid Leukemia |
| 9872/3 | Acute myeloid leukemia with minimal differentiation | Acute Myeloid Leukemia |
| 9873/3 | Acute myeloid leukemia without maturation | Acute Myeloid Leukemia |
| 9874/3 | Acute myeloid leukemia with maturation | Acute Myeloid Leukemia |
| 9891/3 | Acute monoblastic and monocytic leukemia | Acute Myeloid Leukemia |
| 9895/3 | Acute myeloid leukemia with myelodysplasia-related changes ( | Acute Myeloid Leukemia |
| 9896/3 | Acute myeloid leukemia, t(8;21)(q22;q22) RUNX1-RUNX1T1 | Acute Myeloid Leukemia |
| 9897/3 | Acute myeloid leukemia with t(9;11)(p22;q23);MLLT3-MLL | Acute Myeloid Leukemia |
| 9898/3 | Myeloid leukemia associated with Down Syndrome | Acute Myeloid Leukemia |
| 9910/3 | Acute megakaryoblastic leukemia | Acute Myeloid Leukemia |
| 9911/3 | Acute myeloid leuk (megakaryoblastic) with t(1;22)(p13;q13); RBM15-MKL1 | Acute Myeloid Leukemia |
| 9920/3 | Therapy-related myeloid neoplasms | Acute Myeloid Leukemia |

**Table S2**. Raw counts and percentages of early death per year among 16,153 AYAs diagnosed with acute leukemia between the years 2006 to 2020 identified in the SEER database

| **Year of Diagnosis** | Total, n | Early Death, n (%, 95% Confidence Interval) |
| --- | --- | --- |
| 2006 | 974 | 85 (8.7, 7.1-10.7) |
| 2007 | 1004 | 70 (7.0, 5.5-8.7) |
| 2008 | 1017 | 83 (8.2, 6.6-10.0) |
| 2009 | 974 | 72 (7.4, 5.9-9.2) |
| 2010 | 1082 | 66 (6.1, 4.8-7.7) |
| 2011 | 1053 | 70 (6.6, 5.3-8.3) |
| 2012 | 1097 | 66 (6.0, 4.7-7.6) |
| 2013 | 1063 | 53 (5.0, 3.8-6.5) |
| 2014 | 1156 | 57 (4.9. 3.8-6.3) |
| 2015 | 1116 | 56 (5.0, 3.9-6.5) |
| 2016 | 1097 | 62 (5.7, 4.4-7.2) |
| 2017 | 1109 | 55 (5.0, 3.8-6.4) |
| 2018 | 1145 | 51 (4.5, 3.4-5.8) |
| 2019 | 1210 | 63 (5.2, 4.1-6.6) |
| 2020 | 1056 | 66 (6.3, 4.9-7.9) |
